# Supplementary material for: Enhancing Lithium and Sodium Storage Properties of TiO2(B) Nanobelts by Doping with Nickel and Zinc
Source: Nanomaterials (Basel). 2021 Jun 28;11(7):1703. doi: 10.3390/nano11071703 (PMC8306191; doi:10.3390/nano11071703)
Supplement: Supplementary file 1 [file nanomaterials-11-01703-s001.zip › nanomaterials-1273022-supplementary.pdf]

nanomaterials

Enhancing Lithium and Sodium Storage Properties of TiO_2(B) Nanobelts by Doping with Nickel and Zinc

Denis P. Opra *, Sergey V. Gnedenkov, Sergey L. Sinebryukhov, Andrey V. Gerasimenko, Albert M. Ziatdinov, Alexander A. Sokolov, Anatoly B. Podgorbunsky, Alexander Yu. Ustinov, Valery G. Kuryavyi, Vitaly Yu. Mayorov, Ivan A. Tkachenko and Valentin I. Sergienko

Institute of Chemistry, Far Eastern Branch of the Russian Academy of Sciences, 690022 Vladivostok, Russia; svg21@hotmail.com (S.V.G.); sls@ich.dvo.ru (S.L.S.); gerasimenko@ich.dvo.ru (A.V.G.); ziatdinov@ich.dvo.ru (A.M.Z.); alexsokol90@mail.ru (A.A.S.); pab@ich.dvo.ru (A.B.P.); all_vl@mail.ru (A.Y.U.); kvg@ich.dvo.ru (V.G.K.); 024205@inbox.ru (V.Y.M.); tkachenko@ich.dvo.ru (I.A.T.); sergienkovi@yandex.ru (V.I.S.)

* Correspondence: dp.opra@ich.dvo.ru; Tel.: +7-(423)-2311889

For Rietveld refinement the XRD patterns were recorded at a temperature of 293 ± 2 K on a SmartLab diffractometer from the Rigaku (9 kW rotating anode, CuK_α-irradiation, silicon zero-background specimen holder, with a step size of 0.01° on 2θ) in the Bragg Brentano geometry, using a HyPix-3000 detector (1D measurement mode, Ni-filter) for TO-Zn-02 product, and scintillation detector (graphite monochromator) for other samples [1].

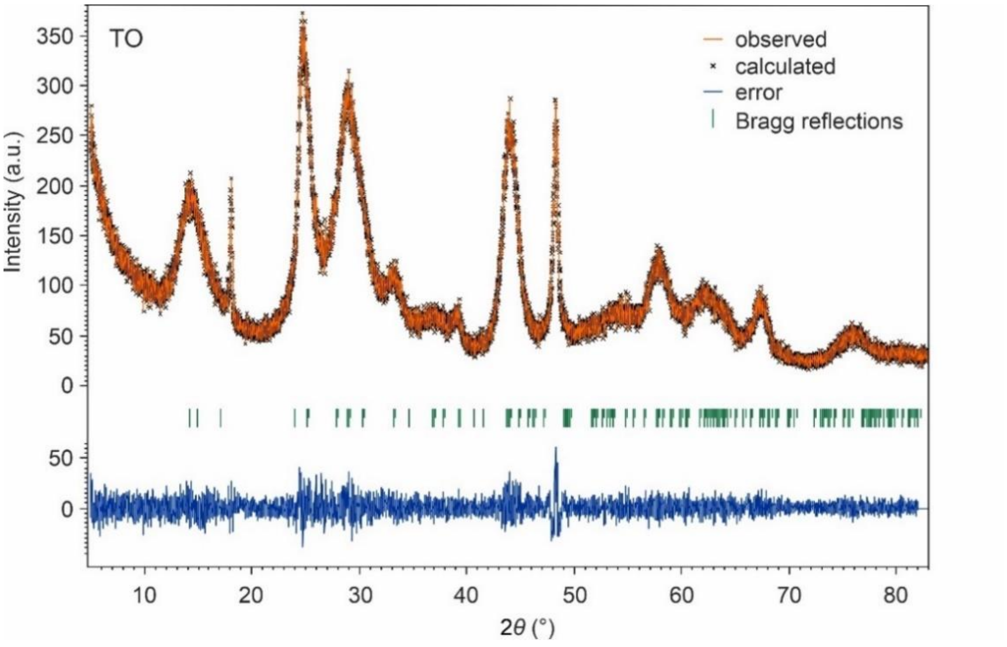

(**a**)

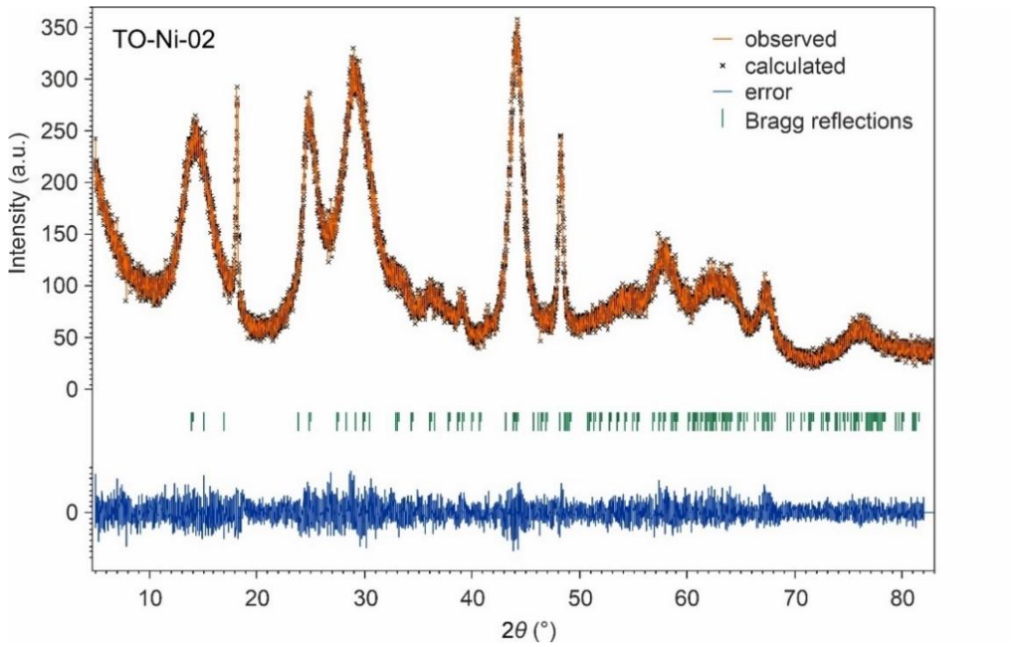
TO-Ni-02
observed
calculated
error
Bragg reflections
Intensity (a.u.)
2θ (°)

(**b**)

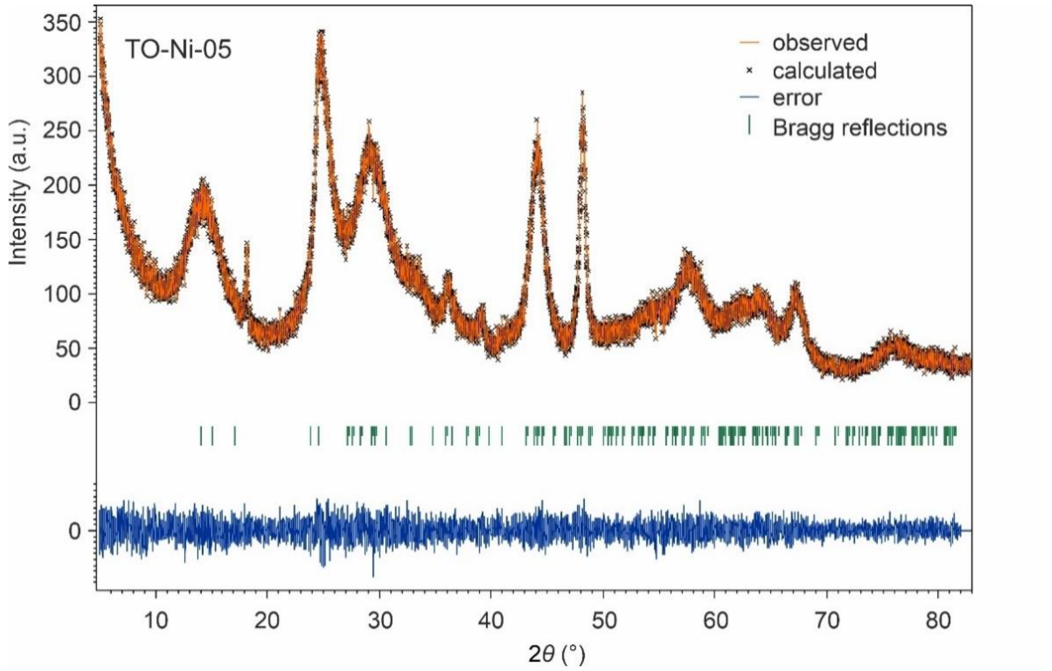
TO-Ni-05
observed
calculated
error
Bragg reflections
Intensity (a.u.)
2θ (°)

(**c**)

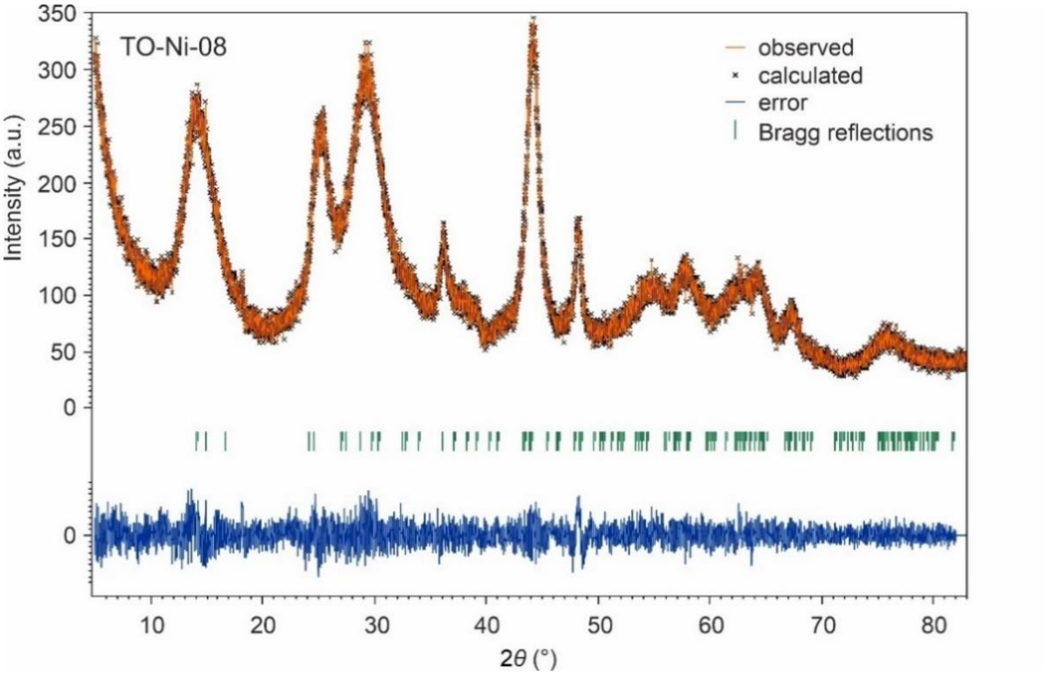

(**d**)

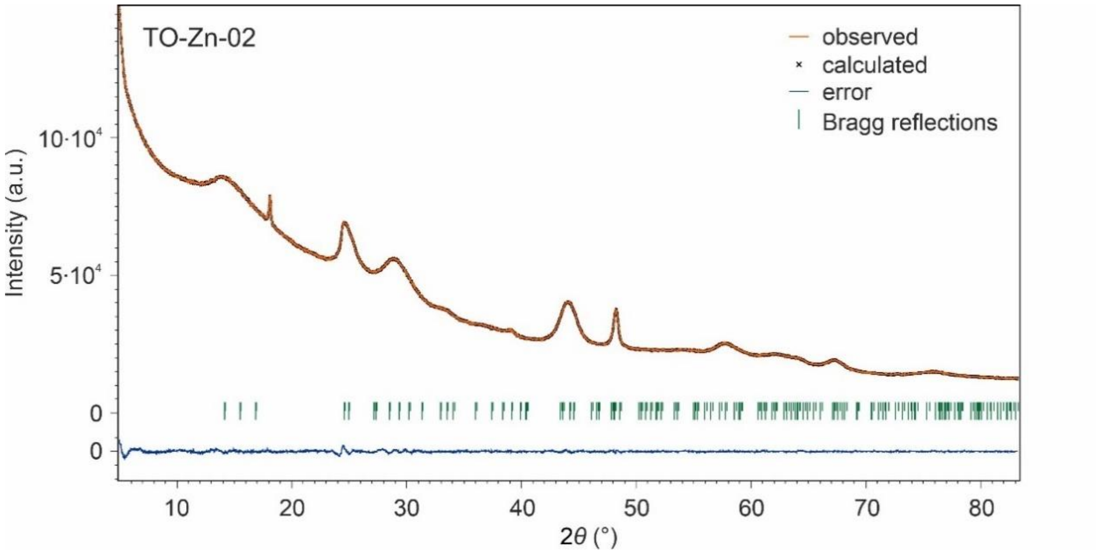

(**e**)

Figure S1. Rietveld plots for (**a**) unmodified TiO_2(B), (**b**) TO-Ni-02, (**c**) TO-Ni-05, (**d**) TO-Ni-08, and (**e**) TO-Zn-02 samples (experimental (—), calculated (×), and difference (—) curves; the vertical bars (|) correspond to the positions of the Bragg reflections).

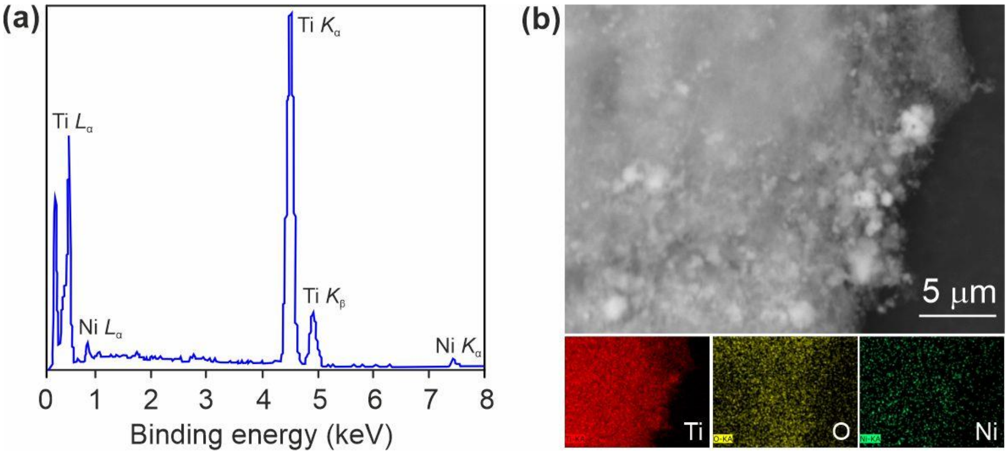

Figure S2. EDX-spectrum **(a)** and maps of elements **(b)** for TO-Ni-05 material.

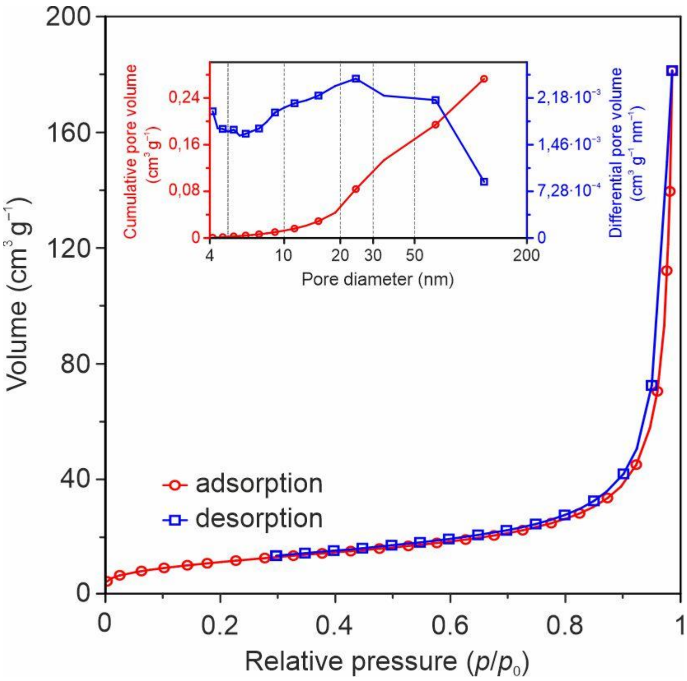

Figure S3. N_2 adsorption–desorption isotherms, cumulative pore volume curve and pore size distribution (inset) for the TO sample.

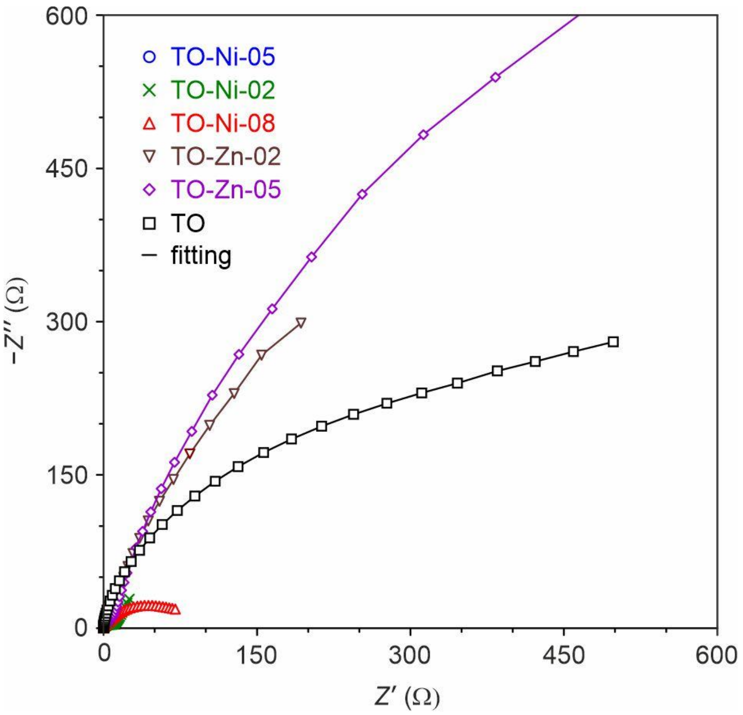

Figure S4. Nyquist diagrams (full-scale view) for the pellets from undoped, Ni- and Zn-modified TiO_2(B) powders.

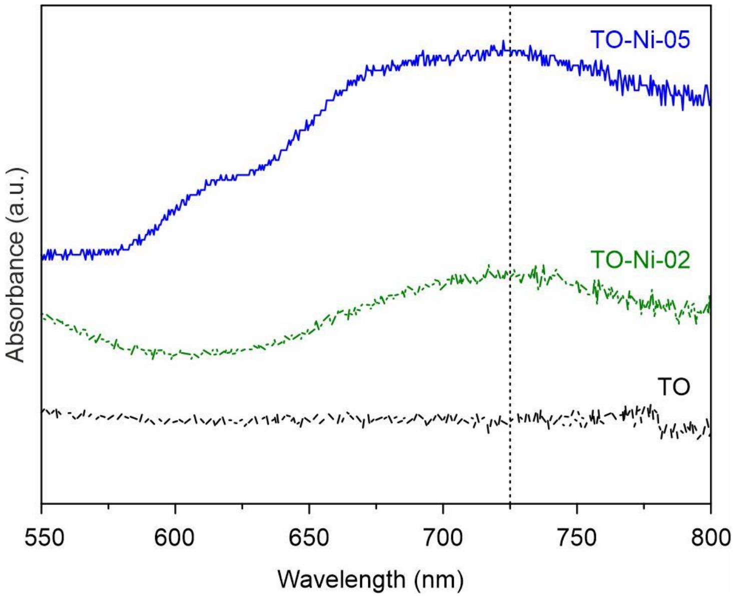

Figure S5. UV–Vis absorption spectra for TO, TO-Ni-02 and TO-Ni-05 materials in the wavelength range of 550–800 nm.

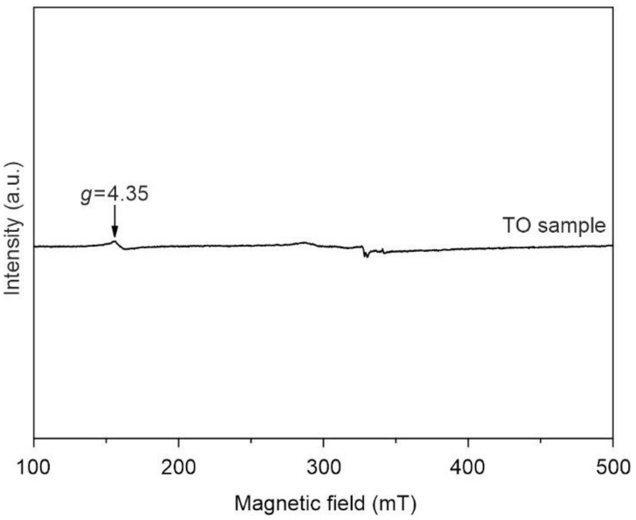

Figure S6. EPR-spectra of undoped bronze TiO_2 nanobelts (TO sample).

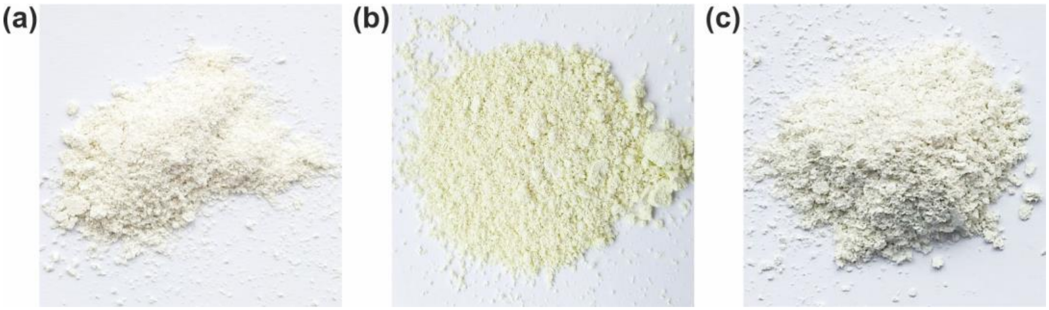

Figure S7. The color of unmodified **(a)**, Ni- **(b)** and Zn-doped **(c)** TO_2(B) samples.

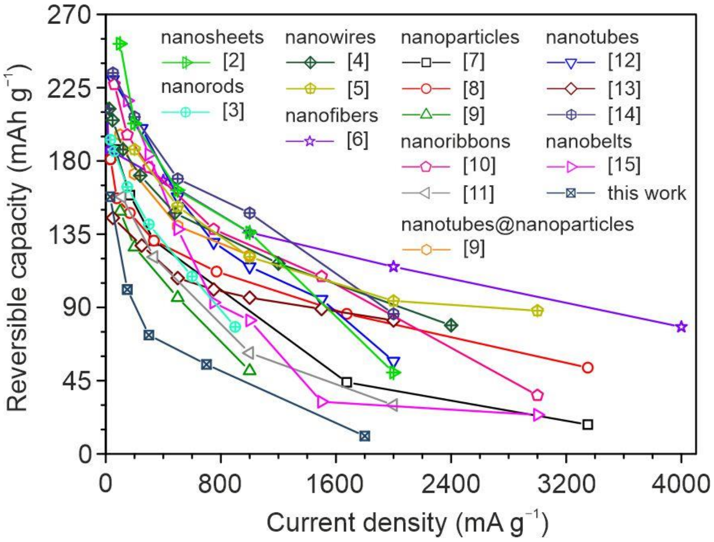

Figure S8. Comparing the rate capability for different TiO_2(B) nanostructures.

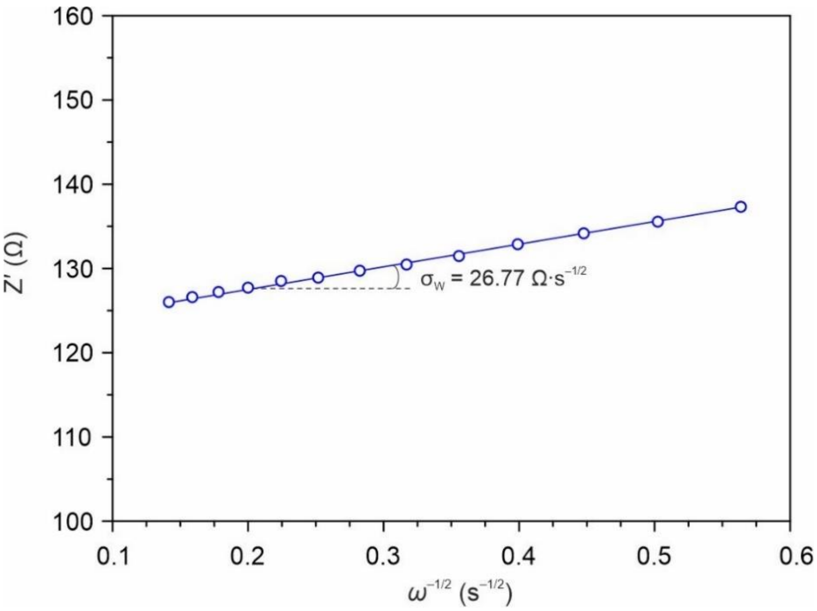

Figure S9. Relationship between Z′ and $\omega^{-1/2}$ in low-frequency region for TO-Ni-05 employed as SIBs anode

Table S1. Rietveld refinement results from XRD data of the undoped, Ni- and Zn-doped TiO_2(B) samples.

Sample	*a* (Å)	*b* (Å)	*c* (Å)	*β* (°)	*V* (Å³)	R_{wp} [a]	R_p [b]	GOF [c]
**TO**	12.393(2)	3.6863(9)	6.505(1)	108.85(2)	281.23(9)	0.0788	0.0601	1.07
**TO-Ni-02**	12.344(4)	3.730(2)	6.634(2)	108.87(4)	289.0(1)	0.0719	0.0549	1.04
**TO-Ni-05**	12.269(4)	3.795(1)	6.601(2)	108.33(3)	291.8(1)	0.0715	0.0548	1.03
**TO-Ni-08**	12.439(4)	3.779(1)	6.571(2)	110.30(2)	289.7(2)	0.0721	0.0548	1.07
**TO-Zn-02**	12.219(1)	3.7486(4)	6.6983(6)	111.058(8)	286.31(4)	0.0069	0.0052	0.98

[a] Weighted profile residual. [b] Profile residual. [c] Goodness of fit.

Table S2. Surface area of materials shown in Figure S6 and features of their fabrication and testing as LIBs electrodes.

TiO_2(B) nanostructure	Specific surface area (m^2 g^{-1})	Carbon additive in electrode (wt.%)	Potential range (V)	Reference (Year)
**nanosheets**	66	45	1.2–2.4	[2] (2012)
**nanorods**	34	13	1–3	[3] (2013)
**nanowires**	25	10	1–2.5	[4] (2013)
	–	18	1–3	[5] (2005)
**nanofibers**	32	10	1–3	[6] (2013)
**nanoparticles**	200	20	1–3	[7] (2011)
	139	10	1–2.5	[8] (2016)
	44	10	1–3	[9] (2015)
**nanoribbons**	–	15	1–3	[10] (2014)
	140	24	1.25–2.5	[11] (2010)
**nanotubes**	–	18	1–2.5	[12] (2012)
	212	18	1–3	[13] (2014)
	36	18	1–2.5	[14] (2006)
**nanotubes@nanoparticles**	> 410	10	1–3	[9] (2015)
**nanobelts**	48	10	0–3	[15] (2013)
	40	13	1–3	this work

Table S3. Measured EIS parameters for TO-Ni-05 electrode in sodium half-cell.

R_s (Ω)	R_f (Ω)	R_{ct} (Ω)	σ_w (Ω $s^{-1/2}$)	D_{Li} (cm^2 s^{-1})
6.07	14.84	104.3	26.77	$6.84 \cdot 10^{-13}$

Table S4. The chemical diffusion coefficient of sodium ions for different Ti-based oxide materials for SIBs anodes.

Material and synthesis method	Morphology features	D_{Na} (cm^2 s^{-1})	Technique of D_{Na} determination	Electrolyte	Reference and year
Ni-doped TiO_2(B), hydrothermal	Mesoporous nanobelts with a width of 40–160 nm and thickness of 3–7 nm	$6.84 \cdot 10^{-13}$	EIS (desodiated; after 5th cycle)	1 M $NaClO_4$ in PC with 5 vol.% FEC	This work
Zn^{2+} doped anatase TiO_2/C, template-assisted coprecipitation	Nanoparticles	$1.33 \cdot 10^{-13}$	EIS (desodiated; after 3th cycle)	1 M $NaPF_6$ in EC/DMC (1:1, v/v)	[16], 2017

N-doped anatase TiO_2–C, electrospinning	Mesoporous nanofibers with a diameter of 100–130 nm	$5.8 \cdot 10^{-13}$	EIS	1 M $NaClO_4$ in PC/EC (1:1, v/v)	[17], 2018
Nb-doped anatase TiO_2, sol-gel	Nanoparticles of ca. 30 nm	$4.31 \cdot 10^{-13}$	EIS (0.3 V; 2nd sodiation)	1 M $NaClO_4$ in EC/DEC (1:1, v/v)	[18], 2015
Anatase/TiO_2(B) heterostructure, hydrothermal	Nanosheet-constructed porous flowers	10^{-15}	GITT (0–2.5 V)	0.6 M $NaClO_4$ in EC/DMC with 5 vol.% FEC	[19], 2019
N-doped rutile TiO_2/C, mechanochemical combined with carbon precursor-assisted calcination	Nanoparticles of ca. 5–50 nm	$2.68 \cdot 10^{-13}$	EIS (0.8 V; 1st desodiation)	1 M $NaClO_4$ in PC with 5 wt.% FEC	[20], 2017
Nb-doped $Na_2Ti_3O_7$, sol-gel	Nanorods with a cross-section size of 250×90 nm	$7.44 \cdot 10^{-15}$	EIS (prior cycling)	1 M $NaClO_4$ in EC/DEC (1:1, v/v)	[21], 2017
Ti^{3+} self-doped $Na_2Ti_3O_7$, sol-gel	Microparticles	$2.37 \cdot 10^{-13}$	EIS (desodiated; after 1st cycle)	1 M $NaClO_4$ in EC/PC (1:1, v/v)	[22], 2018
F-doped $Na_2Ti_3O_7$, solid-phase	Nanorods with a diameter of 100 nm	$1.7 \cdot 10^{-8}$	CV (peak current)	1 M $NaClO_4$ in DEC/DMC (1:1, v/v)	[23], 2018
C-coated $Na_2Ti_3O_7$/$Na_2Ti_6O_{13}$ hybrid, hydrothermal followed by annealing in presence of carbon precursor	Belts with a width of 200–300 nm and thickness of 150 nm	10^{-14}	GITT (0.005–2.5 V; 2nd cycle)	1 M $NaClO_4$ in PC/EC/DMC (4.5:4.5:1, v/v/v)	[24], 2019
$Na_2Ti_3O_7$/$Na_2Ti_6O_{13}$ hybrid	Microrods	10^{-14}	GITT (0.005–2.5 V; 1st desodiation)	1 M $NaClO_4$ in EC/PC (1:1, v/v) with 2 wt.% FEC	[25], 2018
$Na_2Ti_3O_7$@C, hydrothermal coupled with carbon precursor-assisted calcination	Mesoporous cross-linked nanoribbons	$3.17 \cdot 10^{-12}$	EIS	1 M $NaCF_3SO_3$ in diglyme	[26], 2019
$Na_2Ti_3O_7$@C, hydrothermal	Porous nanotubes with an outside diameter of 10 nm	$3.14 \cdot 10^{-13}$	CV (peak current)	1 M $NaClO_4$ in EC/DEC (1:1, v/v) with 10 wt.% FEC	[27], 2017
F-doped $NaTi_2(PO_4)_3$/C, sol-gel	Nanoparticles of ca. 15–20 nm	$1.17 \cdot 10^{-11}$	EIS	1 M $NaClO_4$ in EC/DEC (1:1, v/v) with 2 wt.% FEC	[28], 2019
Mn-doped $NaTi_2(PO_4)_3$/C, solvothermal followed by centrifugation/annealing	Mesoporous nanocrystals of ca. 50–200 nm	$5.39 \cdot 10^{-13}$	EIS (desodiated; after 1000th cycle)	1 M $NaClO_4$ in EC/PC (1:1, v/v)	[29], 2020
$Na_2VTi(PO_4)_3$, sol-gel	Nanoparticles of ca. 200 nm	$2.12 \cdot 10^{-10}$ (anodic) $2.19 \cdot 10^{-10}$ (cathodic)	CV (peak current)	1 M $NaClO_4$ in EC/PC (1:1, v/v)	[30], 2017

C-coated $NaTi_2(PO_4)_3$, solvothermal	Porous nanocubes of ca. 100 nm	10^{-9}–10^{-13}	GITT (1.5–3 V)	1 M $NaClO_4$ in EC/PC (1:1, v/v)	[31], 2019
C-coated $NaTi_2(PO_4)_3$ embedded in the C matrix, sol-gel	Nanoparticles of ca. 30–50 nm dispersed inside the amorphous carbon	$1.8\cdot10^{-10}$ (anodic) $2.32\cdot10^{-10}$ (cathodic)	CV (peak current)	1 M $NaClO_4$ in EC/PC (1:1, v/v)	[32], 2016

Abbreviations are used: ethylene carbonate (EC), propylene carbonate (PC), diethyl carbonate (DEC), dimethyl carbonate (DMC), fluoroethylene carbonate (FEC).

References

1. Rigaku Corp. SmartLab Software 2015.
2. Jang, H.; Suzuki, S.; Miyayama, M. Synthesis of open tunnel-structured TiO_2(B) by nanosheets processes and its electrode properties for Li-ion secondary batteries. *J. Power Sources* **2012**, *203*, 97–102, doi:10.1016/j.jpowsour.2011.11.078.
3. Aravindan, V.; Shubha, N.; Cheah, Y.L.; Prasanth, R.; Chuiling, W.; Prabhakar, R.R.; Madhavi, S. Extraordinary long-term cycleability of TiO_2-B nanorods as anodes in full-cell assembly with electrospun PVdF-HFP membranes. *J. Mater. Chem. A* **2013**, *1*, 308–316, doi:10.1039/C2TA00078D.
4. Shin, K.; Kim, H.J.; Choi, J.-M.; Choi, Y.-M.; Song, M.S.; Park, J.H. Controlled synthesis of skein shaped TiO_2–B nanotube cluster particles with outstanding rate capability. *Chem. Commun.* **2013**, *49*, 2326, doi:10.1039/c3cc38994d.
5. Armstrong, A.R.; Armstrong, G.; Canales, J.; Bruce, P.G. TiO_2–B nanowires as negative electrodes for rechargeable lithium batteries. *J. Power Sources* **2005**, *146*, 501–506, doi:10.1016/j.jpowsour.2005.03.057.
6. Guo, Z.; Dong, X.; Zhou, D.; Du, Y.; Wang, Y.; Xia, Y. TiO_2(B) nanofiber bundles as a high performance anode for a Li-ion battery. *RSC Adv.* **2013**, *3*, 3352, doi:10.1039/c2ra23336c.
7. Wessel, C.; Zhao, L.; Urban, S.; Ostermann, R.; Djerdj, I.; Smarsly, B.M.; Chen, L.; Hu, Y.-S.; Sallard, S. Ionic-liquid synthesis route of TiO_2(B) nanoparticles for functionalized materials. *Chem. - A Eur. J.* **2011**, *17*, 775–779, doi:10.1002/chem.201002791.
8. Li, X.; Li, M.; Liang, J.; Wang, X.; Yu, K. Growth mechanism of hollow TiO_2(B) nanocrystals as powerful application in lithium-ion batteries. *J. Alloys Compd.* **2016**, *681*, 471–476, doi:10.1016/j.jallcom.2016.04.086.
9. Liu, X.; Sun, Q.; Ng, A.M.C.; Djurišić, A.B.; Xie, M.; Liao, C.; Shih, K.; Vranješ, M.; Nedeljković, J.M.; Deng, Z. *In situ* synthesis of TiO_2(B) nanotube/nanoparticle composite anode materials for lithium ion batteries. *Nanotechnology* **2015**, *26*, 425403, doi:10.1088/0957-4484/26/42/425403.
10. Yan, X.; Zhang, Y.; Zhu, K.; Gao, Y.; Zhang, D.; Chen, G.; Wang, C.; Wei, Y. Enhanced electrochemical properties of TiO_2(B) nanoribbons using the styrene butadiene rubber and sodium carboxyl methyl cellulose water binder. *J. Power Sources* **2014**, *246*, 95–102, doi:10.1016/j.jpowsour.2013.07.072.
11. Beuvier, T.; Richard-Plouet, M.; Mancini-Le Granvalet, M.; Brousse, T.; Crosnier, O.; Brohan, L. TiO_2(B) nanoribbons as negative electrode material for lithium ion batteries with high rate performance. *Inorg. Chem.* **2010**, *49*, 8457–8464, doi:10.1021/ic1010192.
12. Qu, J.; Wu, Q.-D.; Ren, Y.-R.; Su, Z.; Lai, C.; Ding, J.-N. Enhanced high-rate performance of double-walled TiO_2-B nanotubes as anodes in lithium-ion batteries. *Chem. - An Asian J.* **2012**, *7*, 2516–2518, doi:10.1002/asia.201200551.
13. Qu, J.; Cloud, J.E.; Yang, Y.; Ding, J.; Yuan, N. Synthesis of nanoparticles-deposited double-walled TiO_2-B nanotubes with enhanced performance for lithium-ion batteries. *ACS Appl. Mater. Interfaces* **2014**, *6*, 22199–22208, doi:10.1021/am505893q.
14. Armstrong, G.; Armstrong, A.R.; Canales, J.; Bruce, P.G. TiO_2(B) nanotubes as negative electrodes for rechargeable lithium batteries. *Electrochem. Solid-State Lett.* **2006**, *9*, A139, doi:10.1149/1.2162327.
15. Huang, H.; Fang, J.; Xia, Y.; Tao, X.; Gan, Y.; Du, J.; Zhu, W.; Zhang, W. Construction of sheet–belt hybrid nanostructures from one-dimensional mesoporous TiO_2(B) nanobelts and graphene sheets for advanced lithium-ion batteries. *J. Mater. Chem. A* **2013**, *1*, 2495, doi:10.1039/c2ta00593j.
16. Li, Y.-N.; Su, J.; Lv, X.-Y.; Long, Y.-F.; Yu, H.; Huang, R.-R.; Xie, Y.-C.; Wen, Y.-X. Zn^{2+} doped TiO_2/C with enhanced sodium-ion storage properties. *Ceram. Int.* **2017**, *43*, 10326–10332, doi:10.1016/j.ceramint.2017.05.063.
17. Nie, S.; Liu, L.; Liu, J.; Xie, J.; Zhang, Y.; Xia, J.; Yan, H.; Yuan, Y.; Wang, X. Nitrogen-doped TiO_2–C composite nanofibers with high-capacity and long-cycle life as anode materials for sodium-ion batteries. *Nano-Micro Lett.* **2018**, *10*, 71, doi:10.1007/s40820-018-0225-1.
18. Zhao, F.; Wang, B.; Tang, Y.; Ge, H.; Huang, Z.; Liu, H.K. Niobium doped anatase TiO_2 as an effective anode material for sodium-ion batteries. *J. Mater. Chem. A* **2015**, *3*, 22969–22974, doi:10.1039/C5TA04876A.
19. Liu, G.; Wu, H.-H.; Meng, Q.; Zhang, T.; Sun, D.; Jin, X.; Guo, D.; Wu, N.; Liu, X.; Kim, J.-K. Role of the anatase/TiO_2(B) heterointerface for ultrastable high-rate lithium and sodium energy storage performance. *Nanoscale Horizons* **2020**, *5*, 150–162, doi:10.1039/C9NH00402E.
20. He, H.; Wang, H.; Sun, D.; Shao, M.; Huang, X.; Tang, Y. N-doped rutile TiO_2/C with significantly enhanced Na storage capacity for Na-ion batteries. *Electrochim. Acta* **2017**, *236*, 43–52, doi:10.1016/j.electacta.2017.03.104.
21. Chen, J.; Zhou, X.; Mei, C.; Xu, J.; Wong, C.P. Improving the sodiation performance of $Na_2Ti_3O_7$ through Nb-doping. *Electrochim. Acta* **2017**, *224*, 446–451, doi:10.1016/j.electacta.2016.12.094.

22. Song, T.; Ye, S.; Liu, H.; Wang, Y.G. Self-doping of Ti^{3+} into $Na_2Ti_3O_7$ increases both ion and electron conductivity as a high-performance anode material for sodium-ion batteries. *J. Alloys Compd.* **2018**, *767*, 820–828, doi:10.1016/j.jallcom.2018.07.186.
23. Chen, Z.; Lu, L.; Gao, Y.; Zhang, Q.; Zhang, C.; Sun, C.; Chen, X. Effects of F-doping on the electrochemical performance of $Na_2Ti_3O_7$ as an anode for sodium-ion batteries. *Materials (Basel).* **2018**, *11*, 2206, doi:10.3390/ma11112206.
24. Hwang, J.; Setiadi Cahyadi, H.; Chang, W.; Kim, J. Uniform and ultrathin carbon-layer coated layered $Na_2Ti_3O_7$ and tunnel $Na_2Ti_6O_{13}$ hybrid with enhanced electrochemical performance for anodes in sodium ion batteries. *J. Supercrit. Fluids* **2019**, *148*, 116–129, doi:10.1016/j.supflu.2019.03.006.
25. Wu, C.; Hua, W.; Zhang, Z.; Zhong, B.; Yang, Z.; Feng, G.; Xiang, W.; Wu, Z.; Guo, X. Design and synthesis of layered $Na_2Ti_3O_7$ and tunnel $Na_2Ti_6O_{13}$ hybrid structures with enhanced electrochemical behavior for sodium-ion batteries. *Adv. Sci.* **2018**, *5*, 1800519, doi:10.1002/advs.201800519.
26. Zhong, W.; Tao, M.; Tang, W.; Gao, W.; Yang, T.; Zhang, Y.; Zhan, R.; Bao, S.-J.; Xu, M. MXene-derivative pompon-like $Na_2Ti_3O_7$@C anode material for advanced sodium ion batteries. *Chem. Eng. J.* **2019**, *378*, 122209, doi:10.1016/j.cej.2019.122209.
27. Li, M.; Xiao, X.; Fan, X.; Huang, X.; Liu, Y.; Chen, L. Carbon coated sodium-titanate nanotube as an advanced intercalation anode material for sodium-ion batteries. *J. Alloys Compd.* **2017**, *712*, 365–372, doi:10.1016/j.jallcom.2017.04.098.
28. Wei, P.; Liu, Y.; Su, Y.; Miao, L.; Huang, Y.; Liu, Y.; Qiu, Y.; Li, Y.; Zhang, X.; Xu, Y.; et al. F-doped $NaTi_2(PO_4)_3$/C nanocomposite as a high-performance anode for sodium-ion batteries. *ACS Appl. Mater. Interfaces* **2019**, *11*, 3116–3124, doi:10.1021/acsami.8b19637.
29. Qu, D.; Chen, Z.; Xu, G.; Liu, X.; Wei, X.; Yang, L. Mesoporous Mn-doped and carbon-coated $NaTi_2(PO_4)_3$ nanocrystals as an anode material for improved performance of sodium-ion hybrid capacitors. *J. Mater. Sci. Mater. Electron.* **2020**, *31*, 17550–17562, doi:10.1007/s10854-020-04310-w.
30. Wang, D.; Bie, X.; Fu, Q.; Dixon, D.; Bramnik, N.; Hu, Y.-S.; Fauth, F.; Wei, Y.; Ehrenberg, H.; Chen, G.; et al. Sodium vanadium titanium phosphate electrode for symmetric sodium-ion batteries with high power and long lifespan. *Nat. Commun.* **2017**, *8*, 15888, doi:10.1038/ncomms15888.
31. Wang, L.; Huang, Z.; Wang, B.; Liu, G.; Cheng, M.; Yuan, Y.; Luo, H.; Gao, T.; Wang, D.; Shahbazian-Yassar, R. Purifying the phase of $NaTi_2(PO_4)_3$ for enhanced Na^+ storage properties. *ACS Appl. Mater. Interfaces* **2019**, *11*, 10663–10671, doi:10.1021/acsami.9b00116.
32. Wang, D.; Liu, Q.; Chen, C.; Li, M.; Meng, X.; Bie, X.; Wei, Y.; Huang, Y.; Du, F.; Wang, C.; et al. NASICON-structured $NaTi_2(PO_4)_3$@C nanocomposite as the low operation-voltage anode material for high-performance sodium-ion batteries. *ACS Appl. Mater. Interfaces* **2016**, *8*, 2238–2246, doi:10.1021/acsami.5b11003.
